# Brain Activity Reflects Subjective Response to Delayed Input When Using an Electromyography-Controlled Robot

**DOI:** 10.3389/fnsys.2021.767477

**Published:** 2021-11-29

**Authors:** Hyeonseok Kim, Yeongdae Kim, Makoto Miyakoshi, Sorawit Stapornchaisit, Natsue Yoshimura, Yasuharu Koike

**Affiliations:** ^1^Institute of Innovative Research, Tokyo Institute of Technology, Yokohama, Japan; ^2^Swartz Center for Computational Neuroscience, Institute for Neural Computation, University of California, San Diego, San Diego, CA, United States; ^3^Department of Industrial Engineering and Economics, Tokyo Institute of Technology, Tokyo, Japan; ^4^Department of Artificial Intelligence, Application Division, EAGLYS Inc., Tokyo, Japan

**Keywords:** electromyography (EMG), electroencephalogram (EEG), delay, subjective response, parietal, robot, robot hand, independent component

## Abstract

In various experimental settings, electromyography (EMG) signals have been used to control robots. EMG-based robot control requires intrinsic parameters for control, which makes it difficult for users to understand the input protocol. When a proper input is not provided, the response time of the system varies; as such, the user’s subjective delay should be investigated regardless of the actual delay. In this study, we investigated the influence of the subjective perception of delay on brain activation. Brain recordings were taken while subjects used EMG signals to control a robot hand, which requires a basic processing delay. We used muscle synergy for the grip command of the robot hand. After controlling the robot by grasping their hand, one of four additional delay durations (0 ms, 50 ms, 125 ms, and 250 ms) was applied in every trial, and subjects were instructed to answer whether the delay was natural, additional, or whether they were not sure. We compared brain activity based on responses (“sure” and “not sure”). Our results revealed a significant power difference in the theta band of the parietal lobe, and this time range included the interval in which the subjects could not feel the delay. Our study provides important insights that should be considered when constructing an adaptive system and evaluating its usability.

## Introduction

In general, when using a human interface system, there are often delays in control, which is an issue that can reduce trust in the utility of the system. For example, time delays in controlling robotic hands are inevitable, leading to lower user satisfaction (Yang and Dorneich, 2015) and performance (Selvidge et al., 2002; Rank et al., 2010). Thus, delays play a critical role in the efficiency of this process. Using questionnaires, several behavioral studies have investigated subjective evaluations of delayed output in specific situations such as first-person shooter games (Quax et al., 2004) and webpage loading (Guse et al., 2015). It was reported that in a real-time cursor control task, subtle and large delays in visual feedback were associated with different brain regions (Kim et al., 2020). Thus, a delay in the general human interface is a key factor that determines the subjective impression of the trustworthiness of the system, which also determines the user experience.

The use of electromyography (EMG) readings as an input to the human-robot interface has been widely used in various applications, including the control of various body parts such as the hands (Yang et al., 2009), fingers (Hussain et al., 2016), and an exoskeleton robot (Lenzi et al., 2012; Peternel et al., 2016). Various methods have been developed to improve the performance of the control, which has been evaluated based on task performance and technological factors (Choi et al., 2009; Al-Timemy et al., 2016; Ao et al., 2017). However, in real-world applications, users are unaware of the EMG activation patterns or the signal processing used to control the robot. Unlike the control using extrinsic parameters such as position, EMG-based control is somewhat counterintuitive for users. Users anticipate real-time movement when controlling the computer cursor or video on the screen; however, in EMG-based control, the control method is rather difficult to understand, and users are unaware of the natural delay. We are not aware when EMG signals start to activate. Thus, in EMG-based control, not only system performance but also subjective usability should be carefully evaluated and investigated. If users cannot control the system, they need to change the muscles that are used to optimize input commands so that the control becomes more efficient. Visual feedback is the most critical information in the process of establishing a reliable input protocol to establish the correct set of muscles that cause a responsive reaction in the robot hand. Although undesirable, confirming a replicable delay is still useful in terms of (meta-level) confirmation of the reliability of the input-output relations. Irregularity in this expected delay makes users skeptical about the effectiveness of the EMG input (“Is my particular use of muscles easily readable to the system?”) and/or reduces trust in the system (“Is this system functioning as expected?”). These negative concerns have a negative impact on user experience. If users do not understand exactly how the system works, this might happen even if the system followed the command configuration well. However, the relationship between the subjective perception of delay and its neural representation under EMG-based robot control is poorly understood. In other words, subjective awareness of the delay may change subsequent neural processes, but this process has rarely been investigated.

To evaluate the influence of the subjective perception of delay on brain activation, we designed an electroencephalogram (EEG) study using EMG-based robot control. We extracted muscle synergy from EMG signals that were used as the grip command of a robot hand that had the necessary basic delay to move. One of four additional delays (0 ms, 50 ms, 125 ms, and 250 ms) was applied in each trial, and subjects were instructed to answer whether the delay was natural or additional or whether they were not sure after they controlled the robot by grasping their hand. We compared the brain activities based on the responses (“sure” and “not sure”).

## Materials and Methods

### Subjects

Nine subjects (six men and three women; mean age ± standard deviation: 26.56 ± 3.17 years) participated in the experiment. All subjects were right-handed and did not have any neurological or motor function disorders. All subjects provided written informed consent prior to the experiment. This study was approved by the ethics committee of the Tokyo Institute of Technology and was conducted in accordance with the Declaration of Helsinki.

### Experimental Apparatus and Data Acquisition

**Figure 1:**
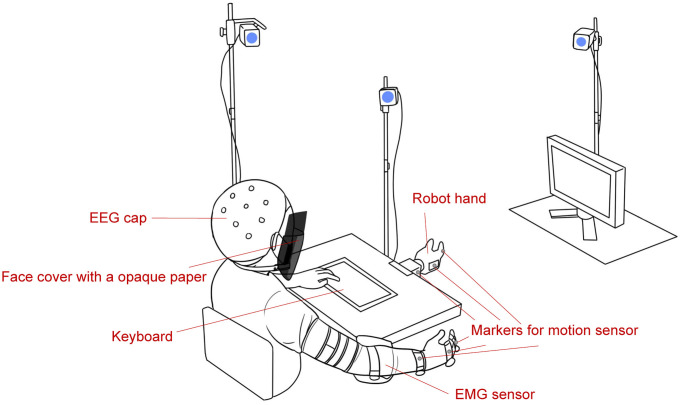
Experimental environment (not to scale). EMG, electromyography; EEG, electroencephalogram.

Figure 1 is a schematic of the experimental environment. During the experiment, the subjects sat on a chair, and their right arm was placed on a supportive surface. They wore an EEG cap, EMG sensor, and markers for motion sensors, and a face cover with a black paper was attached to subjects such that they could not see their right hand. Visual instructions or questions were presented on a screen in front of them, and subjects could make choices by pressing the keyboard button with their left hand. A robot hand (qb SoftHand, qb robotics, Italy) was fixed to a table and was in the subjects’ view. The robot hand and the monitor were placed for subjects to see them without having to move their heads.

Motion data were acquired using the OptiTrack motion capture system (NaturalPoint Inc., Corvallis, OR). Markers were attached to the subjects’ wrist, back of the hand, and middle finger to measure human motion; sensors were placed at the robot’s wrist, back of the hand, and middle finger to measure its motion. We measured EMG signals from 32 channels using an array EMG sensor (Koike et al., 2020) that covers the muscles of the forearm. According to the international 10–20 system (Klem et al., 1999), we measured EEG signals from 64 channels (Fp1, Fp2, Fpz, AF3, AF4, AF7, AF8, AFz, F1, F2, F3, F4, F5, F6, F7, F8, Fz, FT7, FT8, FC1, FC2, FC3, FC4, FC5, FC6, FCz, C1, C2, C3, C4, C5, C6, Cz, T7, T8, TP7, TP8, CP1, CP2, CP3, CP4, CP5, CP6, CPz, P1, P2, P3, P4, P5, P6, P7, P8, P9, P10, Pz, PO3, PO4, PO7, PO8, POz, O1, O2, Oz, and Iz) using the Biosemi ActiveTwo system (Biosemi, Amsterdam, Netherlands).

### Robot Control

Before the actual task, we conducted a calibration session to determine the proper input command of the robot for each subject. Before the calibration began, the subjects placed their right arm on the arm support and relaxed. During calibration, they grasped and opened their hands three times. They were instructed to grip naturally, not exert maximally, and spread their fingers such that their joints did not bend. While they performed the motion, the EMG signals and the angle of the metacarpophalangeal joint of the middle finger were measured. Noisy EMG channels were rejected during the inspection. The remaining EMG channels were rectified and filtered using a second-order Butterworth low-pass filter with a cutoff frequency of 5 Hz. We extracted two muscle synergies from the filtered EMG signals using the HALS algorithm (Cichocki and Phan, 2009). The number of synergies was increased if the synergies were not relevant to hand motion. We used the musculoskeletal model (Kawase et al., 2017) to estimate the joint angle with the corresponding muscle synergies. The estimated joint angle was used as the input command for the robot hand during the task session. After calibration, the subjects practiced long enough to learn how to control the robot that was imitating the human grip. To ensure natural movement control, the robot control was tested immediately after calibration, and recalibration was performed if necessary. We instructed subjects to remember their own way to control the robot and not to change their method during the experiment because we wanted to evaluate how subjects responded to delayed movement compared with the robot with no manipulation of their input commands. In addition, the interval between the human’s movement and the robot’s movement was defined as the response time, where the initiation of movements was detected based on position data. Thus, the response time includes the durations for signal processing and additional delay in some cases.

### Task Description

At the beginning of the experiment, the “wait” message was shown on the screen, instructing subjects to put their right hand on the arm supporter and relax. When their hand was in the resting state, the message was changed into either “natural delay” or “??” When a natural delay was shown on the screen, subjects knew no additional artificial delay would be applied in this trial. Trials showing “natural delay” were set for subjects to know and feel the necessary time required for robot control, which included EMG signal processing and transferring signals to the robot so that they could compare this to when an input command was intentionally delayed in other trials. When “??” was shown on the screen, subjects did not know whether an additional delay would be applied. “additional delay” meant input commands would be intentionally lagged further in addition to a natural delay that is the minimal time required to control the robot for the experiment. When subjects saw either message, they were instructed to look at the robot and control it by grasping their hand and feeling the delay. After the robot had completely gripped, the robot went back to a resting position, and the message was changed into the following: “(1) natural delay; (2) I am not sure; and (3) natural delay + additional delay.” The subjects were instructed to push one of the corresponding buttons (1, 2, or 3) on the keyboard. When subjects felt that the delay was the same as a natural delay or if they knew the delay was natural, they selected 1. When they were not sure whether an additional delay was applied, they selected 2. When they were sure that they felt an additional delay, they selected 3. Further, they were instructed to push either button without thinking and push 2 whenever they found themselves hesitant to select. After making their selections, the participants pushed the space bar to confirm their choice. If subjects pushed the space bar, the message said “Relax,” instructing them to relax until the next trial. This procedure was repeated for all trials.

Each trial had one of four additional delay durations: no delay, 50 ms, 125 ms, and 250 ms. As mentioned above, “natural delay” was displayed on the screen during some trials where no delay was applied and subjects were aware of the nature of the delay before they moved. This was set for subjects so that they do not forget the feeling of a natural delay. Thus, there were five types of trials for each run (one where “natural delay” was shown and four where “??” was shown on the screen). Each run consisted of 24 trials per type of trial where the delay was not indicated (“??”) and 29 trials without an additional delay where the subjects knew a delay was not applied. In each run, the first five trials had no delay, and subjects were informed of this, and the first few trials included additional delays, because if they forgot how the natural delay felt, they would not be able to make a comparison with the trials with additional delay. In total, each run had 125 trials, and we conducted three runs for each subject in the experiment. There was a rest period between the runs.

### EEG Preprocessing

EEGLAB was used for EEG preprocessing. First, EEG signals were resampled at 256 Hz to match the data format for further analysis. Then, the EEG signals were obtained using a high-pass filter with a cutoff frequency of 1 Hz. We used cleanLineNoise (Bigdely-Shamlo et al., 2015) to eliminate line noise and artifact subspace reconstruction for data cleaning (Mullen et al., 2015; Blum et al., 2019). Cleaned signals were re-referenced to an average, and we performed independent component analysis. Each independent component was used to fit an equivalent current dipole model with fit Two Dipoles (Piazza et al., 2016) and identified using ICLabel (Pion-Tonachini et al., 2019).

### Data Analysis

EEG preprocessing was conducted at the single-subject level, whereas analysis from the clustering was conducted at the group level. Independent components, identified as brain components, were used for k-means clustering based on dipole locations. There were 11 clusters, as determined by the silhouette index (Rousseeuw, 1987). We extracted epochs between 0.5 s before the onset of the human’s movement and 2 s after the onset, and we calculated event-related spectral perturbation (ERSP) on independent components related to each cluster using the Morse wavelet (MATLAB Wavelet Toolbox).

For the statistical analysis, trials where delay information (“natural delay” on the screen) was presented were not used. For comparison, three responses (natural delay, not sure, and additional delay) were employed and categorized into two groups, namely, sure (natural and additional delay) and not sure. We combined “natural” and “additional” to identify factors that are not related to the amount of delay but make users feel strange, which could be used to evaluate usability. We thought that we could exclude brain activity related to delay perception by combining both options. In addition, we aimed to help subjects understand the natural delay by providing them with the option to select “natural delay” so that we could exclude the effects of basic delay in controlling. Thus, we provided separate response options (“natural, ” “not sure, ” and “additional”). These options enabled subjects to unconsciously select “not sure” when they do not know whether this delay is “natural” or “additional”. We used time-frequency points between 1 and 50 Hz within an epoch from each cluster for analysis. We used a cluster-based permutation test with weak control of the familywise error rate (Groppe et al., 2011) to determine whether each comparison of the two conditions was statistically significant. The threshold of the *p*-value for preselection was set to 0.01, and the permutation was repeated 5,000 times for each comparison. For comparison based on the response, three subjects were not included because they did not select “not sure” enough much, and the resulting number of samples was not sufficient for analysis.

## Results

**Figure 2:**
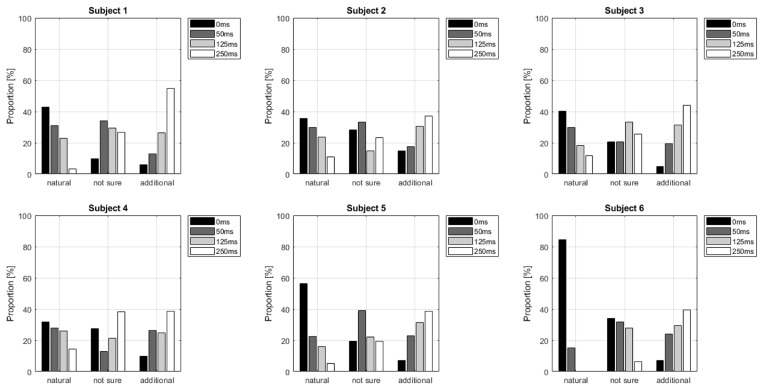
Delay proportion for each response. The summation of the four types of delay is 100% in each response.

**Figure 3:**
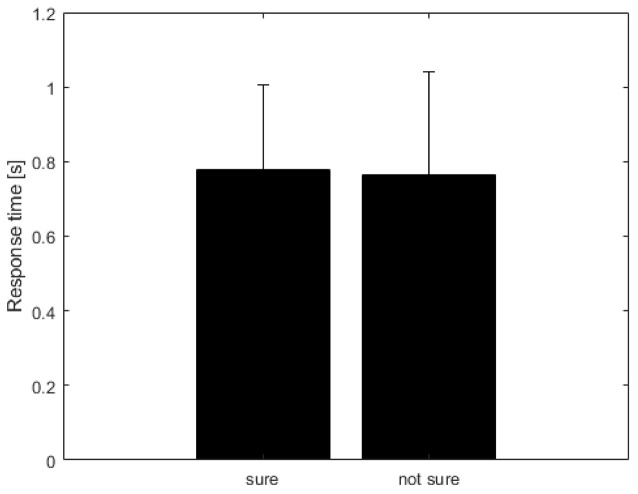
The interval between human movement and robot movement. Unit is seconds (s). The mean value was calculated from six subjects selected for analysis. The error bar represents the standard deviation of the means of subjects.

**Figure 4:**
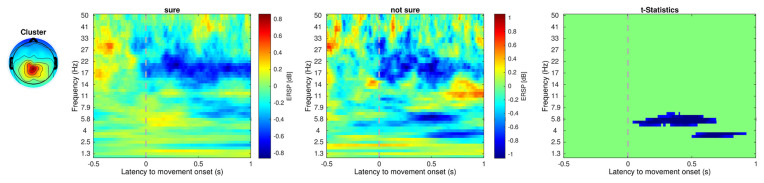
Event-related spectral perturbation of the cluster that showed significant regions within the epoch. Unit is decibel [dB]. The dotted line at 0 s indicates the initiation of the human movement. Cluster 5 includes 21 independent components from six subjects. The power difference (not sure-sure) between about 5–7 Hz was statistically significant.

**Figure 5:**
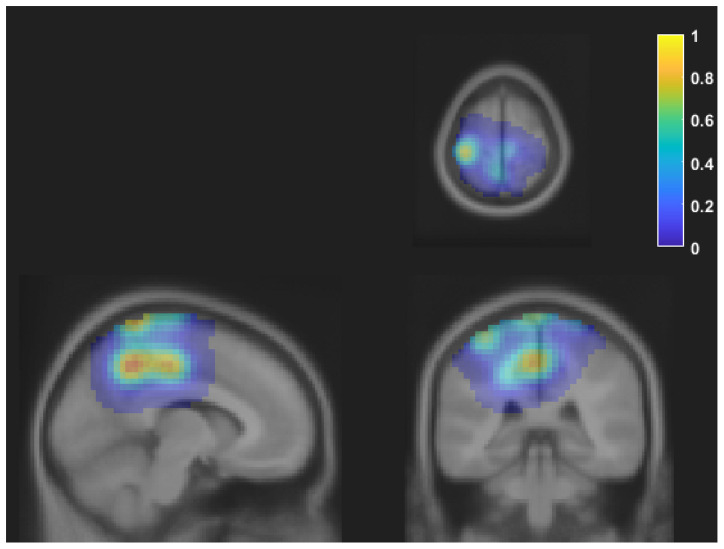
The density of the current dipoles corresponding to each independent component of the cluster. The values were normalized to the maximum value. The cluster includes 21 independent components from six subjects. The mean MNI coordinate was [−9 −35 64], and the standard deviation was [16 14 16]. Each image is shown on axial, sagittal, and coronal planes. Estimated locations of each dipole were the paracentral lobule, postcentral gyrus, and precuneus. MNI, Montreal Neurological Institute.

Table 1 shows the frequency of each response. Figure 2 shows the proportion of behavioral results for each subject. The summation of the four types of delay is 100% in each response. Figure 3 shows the interval between the human’s movement and the robot’s movement. The mean values were about 0.77 s in both cases, and the standard deviation was 0.22 and 0.28, respectively. The difference was not statistically significant (*t*-test; *p* > 0.1). We found a cluster showing significant differences between “sure” and “not sure.” Figure 4 shows the ERSP of the cluster based on the participants’ responses and significantly different masks. A value of 0 s represents the initiation of human movement. We observed a significant power difference (not sure) between 5 and 7 Hz immediately after movement onset. This significant power difference was sustained until 0.7 s. From 0.5 s, a significant power difference of approximately 3.5 Hz was observed. In both cases, a power decrease in the beta band of the cluster was observed from the onset of the subjects’ movement. Figure 5 shows the density of the current dipoles corresponding to each independent component that the cluster consisted of. The cluster was composed of 21 independent components from six subjects. Estimated by the dipoles taking up the head model, anatomical brain regions related to the cluster were the paracentral lobule with a probability of 21%, the postcentral gyrus with a probability of 19%, and the precuneus with a probability of 16%.

**Table 1:** Frequency of each response.

	Natural	Not sure	Additional
S1	145	41	102
S2	101	60	127
S3	147	39	102
S4	161	47	80
S5	94	41	153
S6	39	79	170

## Discussion

In this study, we investigated how subjective delay perception is reflected in the brain using subjects’ responses, regardless of an actual delay. We found that the cluster showing significant differences was associated with the paracentral lobule, postcentral gyrus, and precuneus. The cluster showed significant power differences around 5–7 Hz and 3–4 Hz in the “not sure” condition compared with those in the “sure” condition. The standard deviation of the robot’s response time, which is the interval between human and robot movement, was 0.2–0.3 s in both conditions. This may reflect an individual’s behavior, which includes different ways to input a command to the robot hand.

We observed two significant power differences, at 0–0.7 s and 0.5–0.9 s. This significant power difference could be a reason why the subjects did not have certainty about their response. Uncertainty about subjects’ responses was reflected in the error-related potential, which means that two types of errors can occur (Scheffers and Coles, 2000). Awareness of response errors is also reflected in the brain (Nieuwenhuis et al., 2001). Likewise, awareness of a delay might be different from the delay itself, which could also be reflected in the brain. The length of the delay is associated with a sense of agency and has previously been used to raise a sense of agency (Haering and Kiesel, 2015; Osumi et al., 2019; Bu-Omer et al., 2021). Brain activation related to a sense of agency has been reported in several brain regions, evidenced by a power decrease in the central and bilateral parietal regions (Kang et al., 2015), parieto-occipital regions (Bu-Omer et al., 2021), and pre-supplementary motor areas (Moore et al., 2010). If we consider that the sense of agency refers to a high-level representation that can be related to non-unique neurocognitive phenomena, our results might be related to a sense of agency.

Because our analysis was based on the subjects’ responses, we could consider the cluster in terms of metacognition. A previous work has shown that judgment of agency and judgment of performance were differently related to the brain and can, therefore, be dissociated (Miele et al., 2011). In our experiment, the subjects were instructed to answer whether the delay was natural or additional. They had to identify delays, which could be related to both agency and performance. Another study reported that when subjects felt and did not feel the delay, different activation patterns were observed in the cerebellum, which provided accurate timing information (Leube et al., 2003). This physiological basis, which is not related to actual delay but may influence motor command, supports that delay perception should be considered.

Our significant period included the time range before an input command reached. This suggests that subjects sometimes felt that the robot was not controlled well, regardless of the actual performance of the system. Subjects’ conscious responses might be dominated by their current feeling rather than the actual delay of the system. There have been several studies on brain activity before a stimulus. Pre-stimulus oscillation, in the context of visual perception performance, has also been investigated (Hanslmayr et al., 2007). These results showed that the pre-stimulus alpha power of “perceivers” was lower than that of “non-perceivers.” Additionally, the phases of the alpha at the stimulus onset were related to visual perception (Milton and Pleydell-Pearce, 2016). It has been reported that an increase in pre-stimulus theta oscillations is associated with retrieving successful source memory (Addante et al., 2011), and this phase was associated with the subsequent successful encoding of memory (Cruzat et al., 2021). This evidence may support our results, indicating a significant change in power during the interval when subjects cannot feel a delay.

Previous studies have focused on the stimulus; however, in our study, the users decided the onset of the time. Users expect robots to move and compare to the experience from previous trials, known as trials with natural delay. This situation makes it difficult to understand the results completely, and brain activity dominating perception, such as the pre-stimulus oscillations introduced in previous studies, partially contributed to our results. For EMG-controlled systems, such brain activity dominating perception could be ignored during proper usability evaluation as the user’s response during that trial may not be driven by the actual performance of the system. In addition, this mechanism would be sensitive to brain-machine interfaces requiring human intention, such as the intention to brake when driving a car (Teng et al., 2017), directional intention (Kim et al., 2019), motor imagery intention (Xu et al., 2020), and gait intention (Hasan et al., 2020).

In this study, we have categorized the responses into two groups, namely, “sure” (natural and additional delay) and “not sure”. Out of the nine subjects, only six were considered for analysis as the other three did not select “not sure” enough times. The cluster we found included information for all the subjects but the three subjects’ brain activity during the experiment might not have included the information related to the cluster. Our current experimental paradigm was not optimal to derive “not sure, ” and we did not expect the results obtained in this study. For future studies, the number of subjects should be increased for a better understanding, and a proper experimental paradigm should be designed for investigating our result related to pre-stimulus oscillation.

In this study, we investigated how subjective feelings of delay are reflected in the brain. Our results showed that the power of the theta band in the parietal lobe was significantly changed, suggesting that users might evaluate the system in advance without feeling the response of the system. This should be considered when constructing an adaptive system and evaluating its usability. For further study, delays that can occur when using an EMG interface should be categorized, and the response to each type of delay should be investigated.

## Data Availability Statement

The original contributions presented in the study are included in the article/Supplementary Material, further inquiries can be directed to the corresponding author.

## Ethics Statement

The studies involving human participants were reviewed and approved by the ethics committee of the Tokyo Institute of Technology. The patients/participants provided their written informed consent to participate in this study.

## Author Contributions

HK, NY, and YKo developed the concept and designed the experiment. HK, YKi, and SS acquired the data. HK and MM analyzed the data. HK, YKi, and MM drafted the manuscript. All authors contributed to the article and approved the submitted version.

## Conflict of Interest

SS was employed by the company EAGLYS Inc. The remaining authors declare that the research was conducted in the absence of any commercial or financial relationships that could be construed as a potential conflict of interest.

## Publisher’s Note

All claims expressed in this article are solely those of the authors and do not necessarily represent those of their affiliated organizations, or those of the publisher, the editors and the reviewers. Any product that may be evaluated in this article, or claim that may be made by its manufacturer, is not guaranteed or endorsed by the publisher.

## Abbreviations

EMG: electromyography
EEG: electroencephalogram
ERSP: event-related spectral perturbation.
