# Supplementary figures and images for: Brain Activity Reflects Subjective Response to Delayed Input When Using an Electromyography-Controlled Robot
Source: Front Syst Neurosci. 2021 Nov 29;15:767477. doi: 10.3389/fnsys.2021.767477 (PMC8667890; doi:10.3389/fnsys.2021.767477)

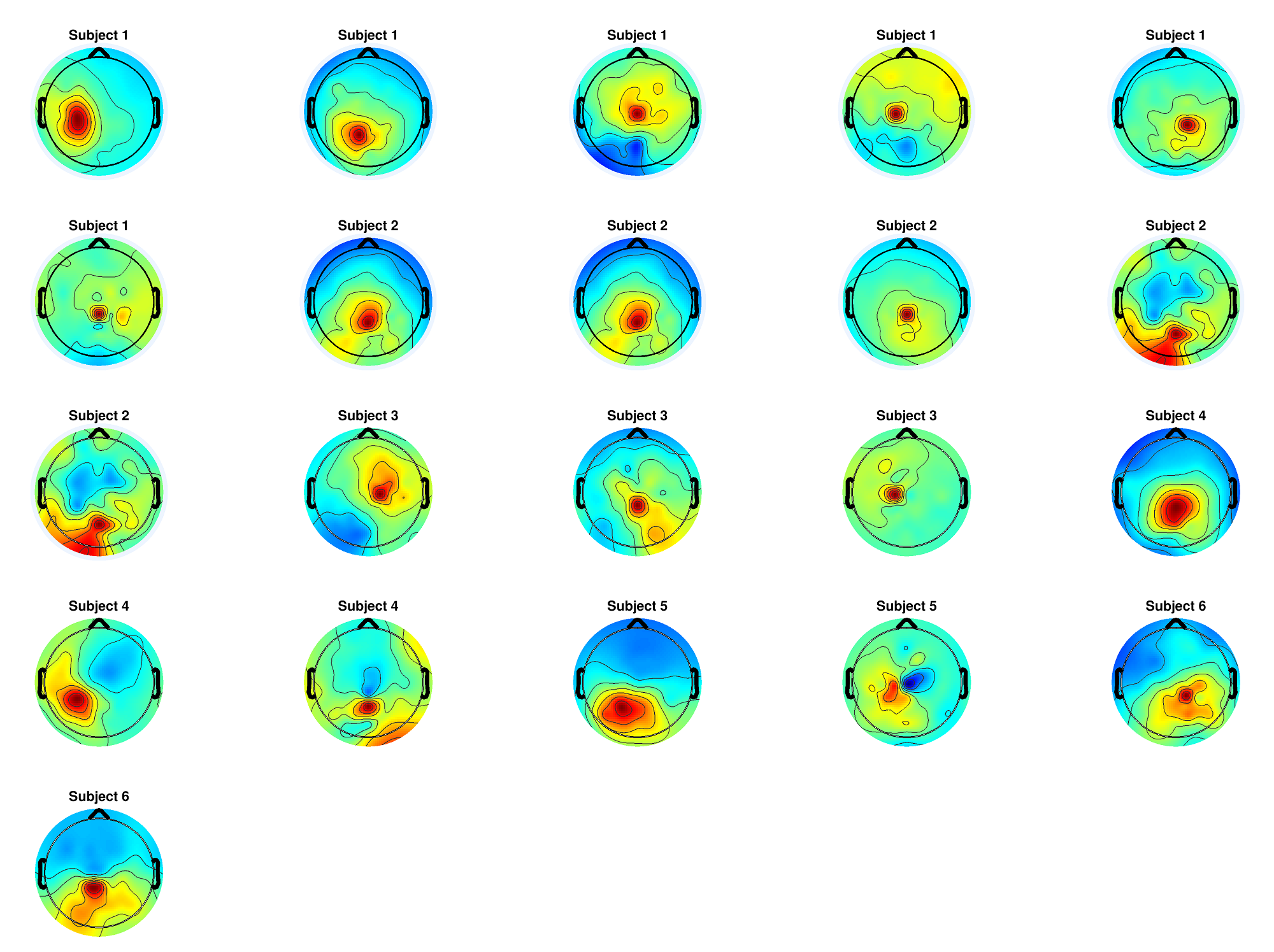

Supplement: Supplementary file 1 [file Image_1.TIF]

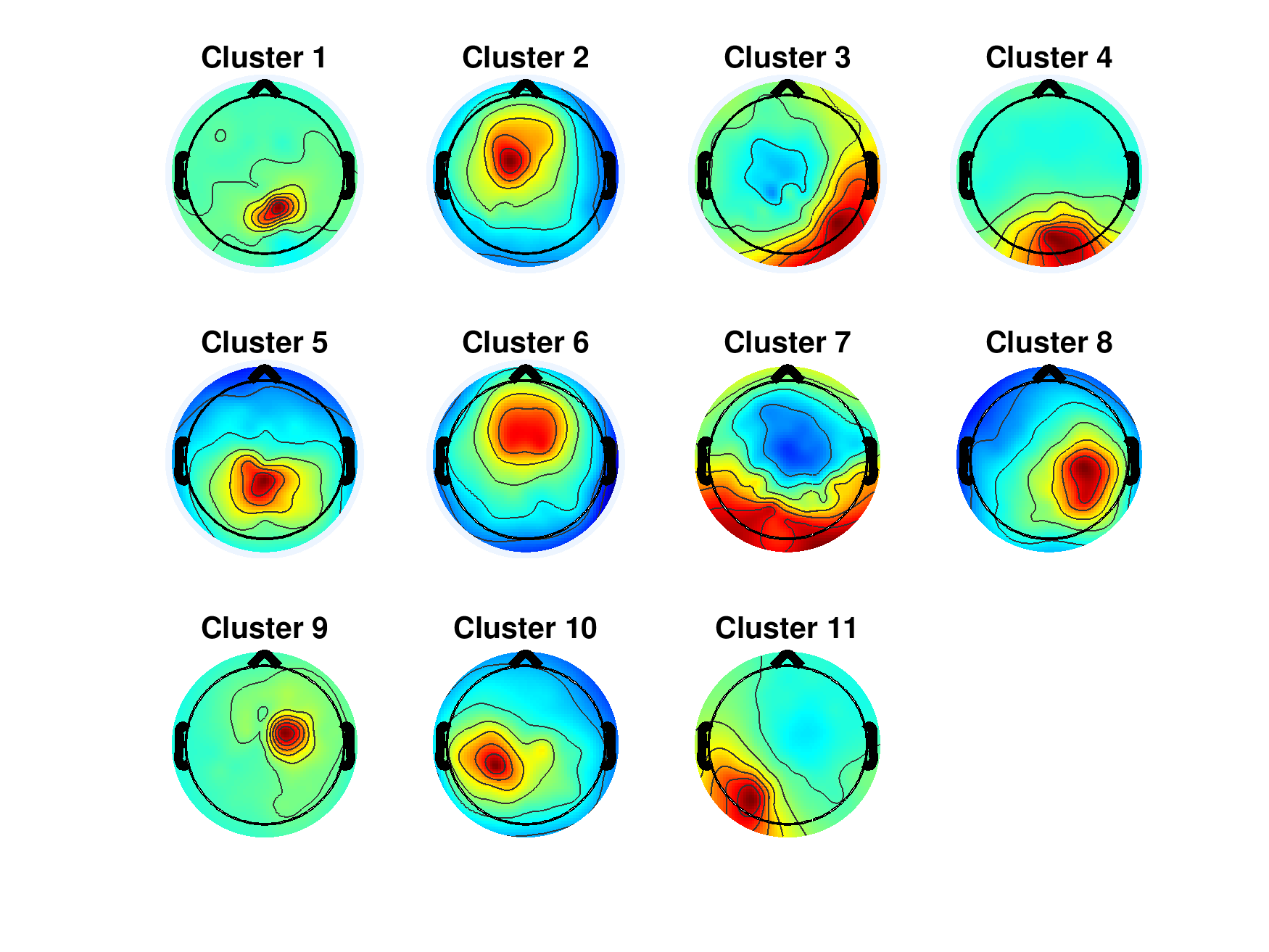

Supplement: Supplementary file 2 [file Image_2.TIF]
